# Early-life stress and the gut microbiome: A comprehensive population-based investigation

**DOI:** 10.1016/j.bbi.2024.02.024

**Published:** 2024-02-23

**Authors:** Rosa H. Mulder, Robert Kraaij, Isabel K. Schuurmans, Carlos Frances-Cuesta, Yolanda Sanz, Carolina Medina-Gomez, Liesbeth Duijts, Fernando Rivadeneira, Henning Tiemeier, Vincent W.V. Jaddoe, Janine F. Felix, Charlotte A.M. Cecil

**Affiliations:** a Department of Child and Adolescent Psychiatry/Psychology, Erasmus MC, University Medical Center Rotterdam, Rotterdam, the Netherlands; b The Generation R Study Group, Erasmus MC, University Medical Center Rotterdam, Rotterdam, the Netherlands; c Department of Internal Medicine, Erasmus MC, University Medical Center Rotterdam, Rotterdam, the Netherlands; d Microbiome, Nutrition & Health Research Unit. Institute of Agrochemistry and Food Technology, Severo Ochoa Centre of Excellence, National Research Council (IATA-CSIC), Valencia, Spain; e Department of Pediatrics, Division of Respiratory Medicine and Allergology, Erasmus MC, University Medical Center Rotterdam, Rotterdam, the Netherlands; f Department of Neonatal and Pediatric Intensive Care, Division of Neonatology, Erasmus MC, University Medical Center Rotterdam, Rotterdam, the Netherlands; g Department of Social and Behavioral Sciences, Harvard T. H. Chan School of Public Health, Boston, MA, USA; h Department of Pediatrics, Erasmus MC, University Medical Center Rotterdam, Rotterdam, the Netherlands; i Department of Epidemiology, Erasmus MC, University Medical Center Rotterdam, Rotterdam, the Netherlands; j Molecular Epidemiology, Department of Biomedical Data Sciences, Leiden University Medical Center, Leiden, the Netherlands

**Keywords:** Microbiome, Early-life stress, Population-based study, Contextual risk, Socio-economic adversity, Alpha diversity, Child development, Tryptophan, Diet

## Abstract

Early-life stress (ELS) has been robustly associated with a range of poor mental and physical health outcomes. Recent studies implicate the gut microbiome in stress-related mental, cardio-metabolic and immune health problems, but research on humans is scarce and thus far often based on small, selected samples, often using retrospective reports of ELS. We examined associations between ELS and the human gut microbiome in a large, population-based study of children.

ELS was measured prospectively from birth to 10 years of age in 2,004 children from the Generation R Study. We studied overall ELS, as well as unique effects of five different ELS domains, including life events, contextual risk, parental risk, interpersonal risk, and direct victimization. Stool microbiome was assessed using 16S rRNA sequencing at age 10 years and data were analyzed at multiple levels (i.e. α- and β-diversity indices, individual genera and predicted functional pathways). In addition, we explored potential mediators of ELS-microbiome associations, including diet at age 8 and body mass index at 10 years.

While no associations were observed between overall ELS (composite score of five domains) and the microbiome after multiple testing correction, contextual risk − a specific ELS domain related to socio-economic stress, including risk factors such as financial difficulties and low maternal education − was significantly associated with microbiome variability. This ELS domain was associated with lower α-diversity, with β-diversity, and with predicted functional pathways involved, amongst others, in tryptophan biosynthesis. These associations were in part mediated by overall diet quality, a pro-inflammatory diet, fiber intake, and body mass index (BMI).

These results suggest that stress related to socio-economic adversity − but not overall early life stress − is associated with a less diverse microbiome in the general population, and that this association may in part be explained by poorer diet and higher BMI. Future research is needed to test causality and to establish whether modifiable factors such as diet could be used to mitigate the negative effects of socio-economic adversity on the microbiome and related health consequences.

## 1 Introduction

Early-life stress (ELS) has been associated with a range of health problems later in life, including psychiatric disorders such as depression and anxiety (Mersky et al., 2013; Nanni et al., 2012), inflammatory and autoimmune diseases (Dube et al., 2009; Kuhlman et al., 2020), as well as cardio-metabolic disorders and all-cause mortality (Chandan et al., 2020; Pierce et al., 2020). Many studies have focused on the role of the stress-regulating hypothalamic−pituitary−adrenal (HPA) axis as a potential underlying biological mechanism (Frodl and O’Keane, 2013; Heim et al., 2008; Kuhlman et al., 2015). Recent findings indicate that the gut and the inhabitant microbiota, which are estimated to include 38 trillion bacterial cells (Sender et al., 2016), may also play a key role in stress regulation through the so-called ‘gut-brain axis’.

The gastrointestinal system is encompassed by the enteric nervous system, which communicates with the central nervous system (Breit et al., 2018; Rhee et al., 2009). The enteric nervous system can produce its own neurotransmitters: for example, an estimated 95 % of serotonin is produced in the gut and enteric nervous system (Foster et al., 2017). Gut microbiota are also directly involved in neurotransmitter production, as several bacterial strains are known to produce serotonin and its precursor tryptophan, as well as other neurotransmitters such as dopamine, noradrenaline, GABA, and acetylcholine (Dinan and Cryan, 2017). At the same time, the gut-brain axis has been linked to stress as well as inflammation (Rea et al., 2016). Multiple lines of evidence point to a cross-talk between the gut microbiome and the immune system. The gut microbiome is part of the first line of defense against pathogens attempting to enter the body via oral ingestion and alterations of the microbiome can induce the production of lymphocytes and inflammatory cytokines (Kamada et al., 2013). In turn, challenges to the immune system can invoke changes to the microbiota composition (Salvo-Romero et al., 2020; Sundman et al., 2017). Such challenges may occur outside of the gut itself, for example, influenza infection in mice has been shown to result in differences in gut microbiome (Ichinohe et al., 2011; Zhang et al., 2020). Furthermore, inflammatory processes stemming from the microbiome can lead to HPA axis activation, whilst stressinduced HPA activation can alter the microbiome through its immunosuppressive functions (Rea et al., 2016). For example, many corticotropin-releasing factor receptors reside in the gut (Stengel and Taché, 2010) and the intestinal mucosa can produce glucocorticoids (Ahmed et al., 2019). Altogether, the gut microbiome has been ascribed a pivotal role in the link between stress, intestinal permeability (a ‘leaky gut’) and (neuro)inflammation (Kelly et al., 2015; Sundman et al., 2017).

Thus far, most evidence on the link between stress and the gut microbiome comes from experimental studies in mice. These studies have shown, for example, that mice exposed to maternal separation and early weaning have a lower diversity of gut microbiota (i.e. α-diversity), as well as differences in the relative composition of the gut microbiota (i. e. β-diversity), compared to control mice (Kemp et al., 2021), and mice exposed to ELS through limited bedding and nesting material showed differential abundance of specific microbial taxa (Reemst et al., 2022). Furthermore, depressive-like behavior induced by maternal separation could be propagated when transferring the microbiota to new mice (De Palma et al., 2015). Microbiota abundance alterations induced by chronic stress have also been shown to be reversed by administration of specific prebiotics, which are dietary fibers that promote a healthy gut flora. These prebiotics were found to reduce stress reactivity, increase tryptophan levels, modify gene expression in the hippocampus and hypothalamus, and reduce chronic stress-induced depressive and anxious behaviors in the mice (Burokas et al., 2017).

Up to this point, a handful of studies have sought to translate studies of ELS and gut microbiota in mice to humans (Callaghan et al., 2020; Coley et al., 2021; Flannery et al., 2020; Hantsoo et al., 2019; Michels et al., 2019; Reid et al., 2021). Several of these have supported an association between higher ELS and lower α-diversity (Callaghan et al., 2020; Michels et al., 2019) as well as differences in microbiome composition, as indicated by β-diversity (Callaghan et al., 2020; Flannery et al., 2020; Reid et al., 2021). Further, ELS was reported to be associated with, amongst others, the abundance of the genera (or taxa within the genera) *Bacteroides* (Flannery et al., 2020; Michels et al., 2019; Reid et al., 2021), *Prevotella* (Hantsoo et al., 2019; Reid et al., 2021), and *Streptococcus* (Flannery et al., 2020; Reid et al., 2021). Last, one study performed an analysis of functional pathway enrichment, reporting that ELS was associated with different microbiome pathways, most prominently a tryptophan metabolic pathway (Flannery et al., 2020).

While these studies provide some preliminary evidence for associations between ELS and the microbiome, others have reported no significant ELS associations with α-diversity (Coley et al., 2021; Hantsoo et al., 2019; Reid et al., 2021), β-diversity (Coley et al., 2021; Hantsoo et al., 2019; Michels et al., 2019), or microbial abundances (Coley et al., 2021). These inconsistencies may be due to a number of factors (Agusti et al., 2023), including (i) the use of small, selected samples − with a median of 32 cases or participants, ranging from target groups such as pregnant women (Hantsoo et al., 2019) to children adopted from or-phanages (Callaghan et al., 2020; Reid et al., 2021) − which can limit the generalizability and comparability of findings; (ii) the use of a cross-sectional design with retrospective measures of ELS in adults, precluding the possibility to examine directionality and potential mediating pathways (Coley et al., 2021; Hantsoo et al., 2019). For example, ELS has been related to a greater preference for high sugar- and fat-containing foods (Torres and Nowson, 2007) and increased risk of obesity (Gunstad et al., 2006), which in turn may affect the microbiome (Dahl et al., 2020; Sweeney and Morton, 2013); (iii) inconsistent adjustment for potential confounders, such as age, sex, or genetic ancestry (Ihekweazu and Versalovic, 2018; Markle et al., 2013; Sisk-Hackworth et al., 2023; Yatsunenko et al., 2012) sometimes while spanning large developmental periods as well (e.g. 5−11 years (Callaghan et al., 2020), or 13−21 years (Reid et al., 2021)); (iv) the focus on ELS as a single entity, such as adoption from orphanages (Callaghan et al., 2020; Reid et al., 2021) or on specific types of stress in isolation, such as stressful life events (Michels et al., 2019), despite evidence that stressors such as stressful life events, socio-economic risk, and direct victimization tend to co-occur (Schuurmans et al., 2022), and as such may exert both shared and unique effects on the microbiome; and (v) the examination of the microbiome at different analytical levels (e.g. global measures of diversity versus abundance of specific microbial taxa), making direct comparison between studies challenging.

To address these issues, we conducted a study examining prospective associations between ELS and the gut microbiome in the general pediatric population, using longitudinal data from over 2,000 children from the Generation R Study. Specifically, we (i) used a comprehensive measure of ELS enabling us to test both specific and overall effects of different ELS domains (i.e. life events, contextual risk, parental risk, interpersonal risk, and direct victimization) from birth to late childhood, (ii) analyzed the gut microbiome at multiple levels (i.e. α- and β-diversity indices, individual genera and enriched functional pathways), and (iii) examined potential mediators of ELS-microbiome associations (i.e. diet and BMI). Based on the existing animal and human literature, we hypothesized that higher stress would associate with lower diversity of the microbiome. No *a-priori* hypothesis was made on which genera or functional pathways would be associated with early-life stress. Exploratory analyses were performed to test mediation of diet-related factors and BMI in ELS-microbiome associations.

## 2 Methods

### 2.1 Study population

The study was embedded in the Generation R Study, a population-based pregnancy cohort in Rotterdam, the Netherlands (Kooijman et al., 2016). The Generation R Study is conducted in accordance with the World Medical Association Declaration of Helsinki and has been approved by the Medical Ethics Committee of Erasmus MC, University Medical Center Rotterdam. Written informed consent was obtained for all participants in accordance with applicable regulations. Information on early life stressors up to age 10 was available in 5,995 children. Microbiome data at 10 years was available for 2,297 of these children. Of these, 150 children were part of a sibling pair or trio. One of each pair or two of each trio of siblings was selected based on data completeness or otherwise randomly selected from the sample, leaving a final sample of 2,004 children.

### 2.2 Early-life stress

Five risk domains of early-life stress experienced between birth and age 10 years (i.e. age of microbiome assessment) were measured: *life events* (e.g. death of a parent or pregnancy complications), *contextual risk* (e.g. financial difficulties or low maternal education), *parental risk* (e.g. criminal record or parental depression), *interpersonal risk* (e.g. family conflicts or loss of a friend) and *direct victimization* (e.g. bullying or harsh parenting) (https://github.com/SereDef/cumulative-ELS-score). Each risk domain represented a cumulative score of 8 to 13 domain-specific risk factors, which were based on one or multiple questionnaire items. Scores within each domain were adjusted for total amount of risk factors (meaning scores are relative and ranged between 0 and 1) and subsequently averaged into a composite score of overall ELS. Previously, this ELS score and its individual domains have been found to associate with neural, cognitive, and mental health outcomes (Bolhuis et al., 2022; Defina et al., 2023; Schuurmans et al., 2022). Missing data (maximum set at 50 % of items; item-level range was 0−32 %) on risk factors were imputed with the *mice* package (Buuren and Groothuis-Oudshoorn, 2010) in R (R Core Team, 2013), using a maximum of 60 iterations, creating 30 datasets (Defina et al., 2023). Finally, the overall ELS score and risk domain scores were standardized within the sample.

### 2.3 Gut microbiome

Stool samples were collected by the participants at home at the age of 10 years (Radjabzadeh et al., 2021), using the Commode Specimen Collection System (Covidien, Mansfield, MA). An aliquot of approximately 1 g was transferred to a 25 × 76 mm collection tube (Minigrip Nederland, Lelystad, The Netherlands) without preserving agent. The samples were dated and sent to the Erasmus MC by In case the sample could not be mailed right after production (i.e. stool production in evening or weekend), participants were asked to store the sample at 4 °C in the home fridge. Upon arrival at Erasmus MC, samples were stored at −20 °C. DNA was isolated with the Automated Arrow Stool DNA isolation kit (Isogen Life Science, de Meern, The Netherlands) after bead beating with 0.1 mm silica beads (MP Biomedicals, LLC, Bio Connect Life Sciences BV, Huissen, The Netherlands). The 309F-806R primer pair was used with dual indexing to amplify the V3 and V4 hypervariable regions of the 16S rRNA gene and sequenced with Illumina MiSeq (Illumina Inc., San Diego, CA) on the V3 flowcell (MiSeq Reagent Kit v3, 2 x 300 bp) at an average depth of 50,000 read-pairs per sample. Raw reads were demultiplexed and tagclearn v0.16 (Schmieder et al., 2010) was used to trim off primers, barcodes and heterogeneity spaces. DADA2 (Callahan et al., 2016) was used for quality filtering (trim = 0, maxEE = c(2,2), truncQ = 2, rm.phix = TRUE) and to denoise, cluster and merge the reads. The SILVA v138.1 rRNA database (Quast et al., 2012) and an RDP naïve Bayesian classifier (Wang et al., 2007) were used to assign a taxonomy to the Amplicon Sequence Variants (ASVs). A phylogenetic tree was constructed with the *phangorn* package (Schliep et al., 2016) in R. Univariate differential abundance and β-diversity analyses were studied at the level of the genera. To this end, samples with <6000 reads were filtered out, and genera with <0.005 % of total number of reads, or with >90 % zero values were filtered out. Genus-level zero values were then imputed using count zero multiplicative imputation with the *zCompositions* R package (Palarea-Albaladejo and Martín-Fernández, 2015) and the data were centered log ration transformed.

### 2.4 Statistical analyses

**Fig. 1:**
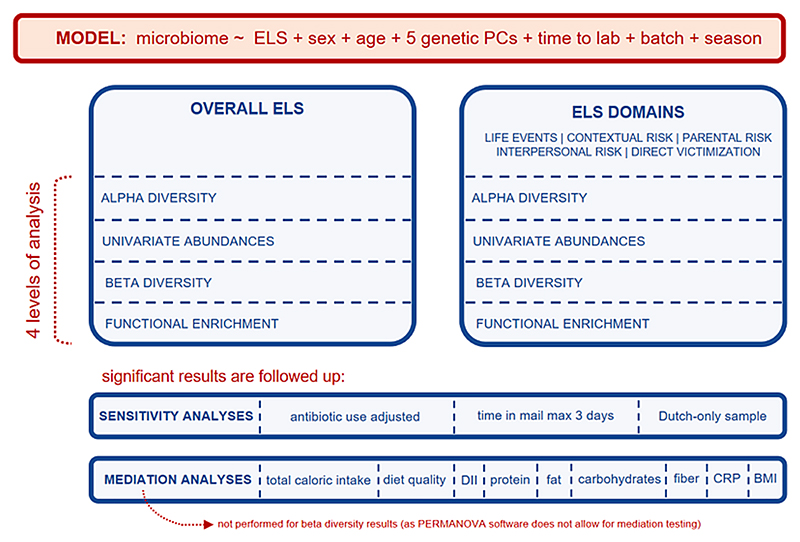
Analysis set-up.

An overview of the analyses conducted is depicted in Fig. 1. Analyses were performed in R, with early-life stress (birth-age 10yrs) modeled as independent variable and the microbiome (age 10yrs) as dependent variable, adjusting for relevant covariates (described below). The microbiome was studied at four different levels:

(i) (i) *α-diversity indices* (richness (Fisher et al., 1943), Shannon diversity (Shannon, 1948), and inverse Simpson index (Simpson, 1949)), as a reflection of overall diversity of observed species, using linear regressions;
(ii) *univariate* (i.e. *per taxon) differential abundances* of the taxa at the genus-level were studied using the *ANCOMB-BC* R package (Lin and Peddada, 2020), for a total of 103 genera. Since there is a plethora of methods of differential abundance analysis available (Nearing et al., 2022) simple univariate robust linear regressions were also performed for the overall ELS score and coefficients were correlated with those of ANCOMB-BC as a sensitivity analysis;
(iii) *β-diversity*, or multivariate abundances, genus-level taxa were examined to study the pair-wise dissimilarities of Euclidian distances between samples to detect clustering of microbiome profiles. Permutational multivariate analysis of variance (PERMANOVA; 999 permutations) was performed with the *vegan* R package (Oksanen et al., 2013), enabling us to quantify variance in the relative composition of the genus-level abundances explained by ELS;
(iv) *functional pathway enrichment* was studied using PICRUSt2 prediction (Douglas et al., 2020) of MetaCyc microbial pathways using the default EPA-NG (Barbera et al., 2019) placement option and MinPath (Ye and Doak, 2009) biological pathways reconstruction based on Enzyme Commission (EC) numbers (Caspi et al., 2018), resulting in 309 pathways.

Each analysis was performed for (i) overall ELS and (ii) the five individual risk domains (mutually adjusted; i.e. entered simultaneously as multiple independent variables). For overall ELS, for each stress domain, and at each level of the microbiome analysis, the significance threshold was corrected for the number of tests with the Benjamini-Hochberg correction (Benjamini and Hochberg, 1995) using a significance cutoff of *q* < 0.05.

Each analysis was adjusted for child sex, age at stool production, 5 genetic principal components (PCs) derived from DNA samples (Medina-Gomez et al., 2015), the time in mail (i.e. number of days between stool sample production and arrival at Erasmus MC, max 5 days), season of stool sample production (autumn, spring, summer, winter), batch variables (DNA isolation batch (*n* = 2) and sequencing run batch (*n* = 3)), and number of sequencing reads (for more details on technical covariates, see Radjabzadeh et al. (2021)).

If an association was found after multiple testing correction (*q* < 0.05), three sensitivity analyses were performed: 1) to test if antibiotic use confounded the relationship, analyses were reran adjusting for past year antibiotic use (four categories: no use/last month/1 to 3 months ago/3 months to a year ago), as reported by the primary caregiver at the time of microbiome sample production, 2) to test if associations were influenced by samples that had spent a longer time to reach the laboratory, analyses were performed in a subset selected for time in mail of maximum 3 days (*n* = 1,592), and 3) to test if associations were modified by ethnicity, we repeated analyses in a subsample of children with parents of Dutch origin, which was the largest ethnic subset (*n* = 1,245).

Missing values on covariates (*n* = 190 for the genetic PCs, *n* = 4 for antibiotic use) were imputed on the full set of independent variables (including covariates), exploratory mediators including diet factors and BMI, as well as alpha diversity measures and variables of maternal education and ethnicity with *mice* (Buuren and Groothuis-Oudshoorn, 2010) in R, using a maximum of 100 iterations for 30 imputed sets. Pooled statistics are reported for analysis (i) and (iv). For the *ANCOM-BC* analysis (ii) and the PERMANOVA analysis of (iii) we use the median summary statistics over 30 imputed sets.

### 2.5 Exploratory analyses of possible mediators

If an association was found, exploratory analyses were performed to test for possible mediators, selected based on previous literature: nutritional intake as defined by overall diet quality (Isasi et al., 2015; Pinart et al., 2021; Yu et al., 2021), the Dietary Inflammatory Index (Alfreeh et al., 2020; Zheng et al., 2020), total caloric intake (Dallman et al., 2007; Zhang et al., 2022), protein, fat, and carbohydrate intake (expressed in energy percentage) (Dahl et al., 2020; Torres and Nowson, 2007), as well as dietary fiber intake (in grams per MJ) (Lin et al., 2011; Morrison et al., 2020), body mass index (BMI) of the child (Bowyer et al., 2018; Isasi et al., 2015; Khaled et al., 2020; Sweeney and Morton, 2013), and inflammatory marker C-reactive protein (CRP) (Johnson et al., 2013; Lin et al., 2016). Nutritional intake variables were measured at age 8 years using a parent-reported Food Frequency Questionnaire (Dutman et al., 2011; van der Velde et al., 2019), which contains 71 food items, selected based on a national food consumption survey in the Netherlands (Nutrition Centre Netherlands, 1998). The diet quality score (van der Velde et al., 2019) was based on adherence to the recommendations for children from the Netherlands Nutrition Center (Brink et al., 2016) and the Dutch Guidelines for a healthy Diet of 2015 (Health Council of the Netherlands, 2015). The Dietary Inflammatory Index was based on 16 pro- and anti-inflammatory food parameters, which were derived from a study of 1,943 primary research articles on food intake and 6 inflammatory biomarkers (interleukin[IL-]-1β, IL-4, IL-6, IL-10, tumor necrosis factor-α, and C-reactive protein), together indicating the (pro-)inflammatory potential of the diet (Khan et al., 2018; Shivappa et al., 2014). BMI was based on weight and height measures at the research center at 10 years (kg/m^2^) and was sex-and age adjusted to SD-scores (BMI SD score) according to the Dutch reference growth curves (Fredriks et al., 2000). High-sensitivity (hs-)CRP was measured from venous blood at age 10, using an immunoturbidimetric assay on the Architect System (Abbot Diagnostics B.V., Hoofddorp, The Netherlands). The within-run precision for hs-CRP was 1.3 % at 12.9 mg/L and 1.2 % at 39.9 mg/L. The lowest level of detection was 0.3 mg/ L. Scores were categorized into 0−1 mg/L, 1−3 mg/L, and > 3 mg/L, to represent normal levels, low grade inflammation levels, and moderate to severe inflammation or acute inflammation levels, respectively (Pearson et al., 2003). The sample size of the of the analyses containing nutrition variables were *n* = 1,594, that of the analysis containing BMI was *n* = 2,003, and that of the analysis containing CRP was *n* = 1,420.

Mediation was tested using Sobel’s test (Preacher and Leonardelli, 2001), in three steps. First, the initial model was repeated in the analytical sub-sample to quantify the total effect of ELS on the microbiome. If the total effect was significant, associations were then tested between ELS and the hypothesized mediators. Last, if an association was found, the mediator was added to the model of stress and the microbiome, to estimate the indirect path of stress via the mediator to the microbiome. Mediation analyses were adjusted for the same covariates as the main analyses. Since the mediation analyses were exploratory, the significance threshold was set at *p* < 0.05.

## 3 Results

Sample characteristics are described in Table 1, correlations in Supplementary Fig. 1. Children were on average 9.8 years old and approximately half (50.4 %) of the sample consisted of boys. For most children (62.1 %) both parents were of Dutch origin, and the sample had a relatively large ethnic variety: of the remaining sample, the largest groups consisted of children with at least one parent born elsewhere in Europe (7.6 %), Surinam (5.7 %), or Africa (5.2 %).

### 3.1 α-diversity

**Fig. 2:**
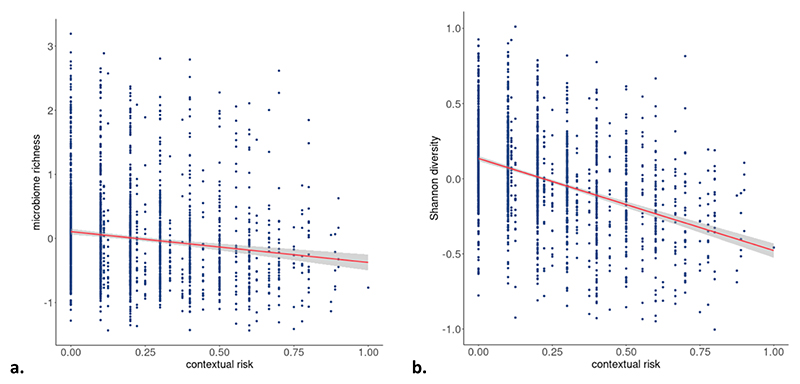
Associations of contextual risk with a) microbiome richness (observed ASV), and b) Shannon diversity, indicated with a 95% confidence interval. The associations are adjusted for the other stress domains (life events, parental risk, interpersonal risk, direct victimization) and covariates (child sex, age, 5 genetic PCs, time in mail, season of production, batch, and number of reads). Results are pooled estimates from 30 imputed datasets.

We found no associations of overall ELS with any of the three α-diversity indices (Table 2). Likewise, we found no unique associations for the stress domains of life events, parental risk, interpersonal risk, or direct victimization with any of the three α-diversity indices. Contextual risk, however, associated negatively, and independently of other stress domains, with microbiome richness (beta = −0.048, 95 % CI = −0.086; −0.011, *q* = 0.031) and Shannon diversity (beta = −0.063, 95 % CI = −0.116; −0.010, *q* = 0.031), after multiple testing correction (Table 2; Fig. 2).

### 3.2 Univariate analysis of differential abundance

We found no associations of overall ELS with abundance of individual genera after multiple testing correction (Supplementary Table 1). A sensitivity analysis using simple robust linear regressions showed similar results (Supplementary Table 2) with estimates highly correlated (*r* = 0.87, *p* = 4.39 × 10^−32^). We also did not find associations between any of the individual ELS domains and the genera (Supplementary Tables 3−7).

### 3.3 β-diversity

Overall ELS was not associated with the relative microbiome composition of the genus-level as indexed by the β-diversity measure (Table 3). Similarly, we found no associations between β-diversity and the stress domains of life events, parental risk, interpersonal risk, or direct victimization. However, a small proportion (0.11 %) of variance in β-diversity could be explained by contextual risk (median F = 2.33, median R^2^ = 0.0011, median *p* = 0.001).

### 3.4 Functional enrichment

**Fig. 3:**
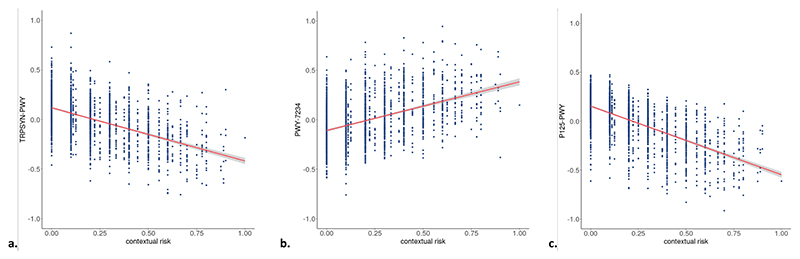
Associations of contextual risk with a) L-tryptophan biosynthesis pathway, b) inosine-5′-phosphate biosynthesis III pathway, and c) (R,R)-butanediol biosynthesis superpathway. The associations are adjusted for the other stress domains (life events, parental risk, interpersonal risk, direct victimization) and covariates (child sex, age, 5 genetic PCs, time in mail, season of production, batch, and number of reads). Results are pooled estimates from 30 imputed datasets.

We found no associations between overall ELS and predicted Meta-Cyc pathways (Supplementary Tables 8−13) after multiple testing correction. Further, no MetaCyc pathway enrichment was found for the stress domains of life events, parental risk, interpersonal risk, or direct victimization, but significant differential enrichment of three pathways was found in association with contextual stress (Table 4, Fig. 3). Specifically, contextual stress was (i) negatively associated with the L-tryptophan biosynthesis pathway (TRPSYN-PWY; B = −1.05 × 10^−04^, 95 % CI = −1.49 × 10^−04^; −6.07 × 10^−05^, *q* = 0.002), (ii) positively associated with the inosine-5′-phosphate biosynthesis III pathway (PWY-7234; B = 8.88 × 10^−05^, 95 % CI = 4.48 × 10^−05^; 1.33 × 10^−04^, *q* = 0.032), and (iii) negatively associated with the (R,R)-butanediol biosynthesis superpathway (P125-PWY; B = −3.27 × 10^−05^, 95 % CI = −5.06 × 10^−05^; −1.48 × 10^−05^, *q* = 0.047).

### 3.5 Sensitivity analyses

Significant associations between the ELS domain of contextual risk and microbiome features (α-diversity [richness and Shannon diversity], β-diversity, and the three functional pathways [L-tryptophan biosyn-thesis pathway, inosine-5′-phosphate biosynthesis III pathway, and (R, R)-butanediol biosynthesis superpathway]) were re-analyzed, in a series of three sensitivity analyses. Across sensitivity analyses (adjusting for antibiotic use, time in mail maximum 3 days, Dutch-only sample), results remained consistent (Supplementary Tables 14−16).

### 3.6 Exploratory mediation analyses

Exploratory mediation analyses were performed for the association of contextual risk with the α-diversity indices (microbiome richness and Shannon diversity) and the three enriched MetaCyc pathways (L-tryptophan biosynthesis pathway, inosine-5′-phosphate biosynthesis III pathway, and (R,R)-butanediol biosynthesis superpathway). Six diet-related factors: diet quality score, total caloric intake, relative protein intake, relative fat intake, relative carbohydrate intake, and relative fiber intake, as well as BMI SD score were tested as possible mediators (see Supplementary Table 17 for inter-correlations). First, in the smaller samples selected for complete data on diet-related factors and BMI, the association between contextual risk and the microbiome was re-tested and confirmed (*p* < 0.05). Second, contextual risk was found to associate with all hypothesized mediators (*p* < 0.05) except relative carbohydrate intake, hence this variable was not considered further as a mediator.

**Fig. 4:**
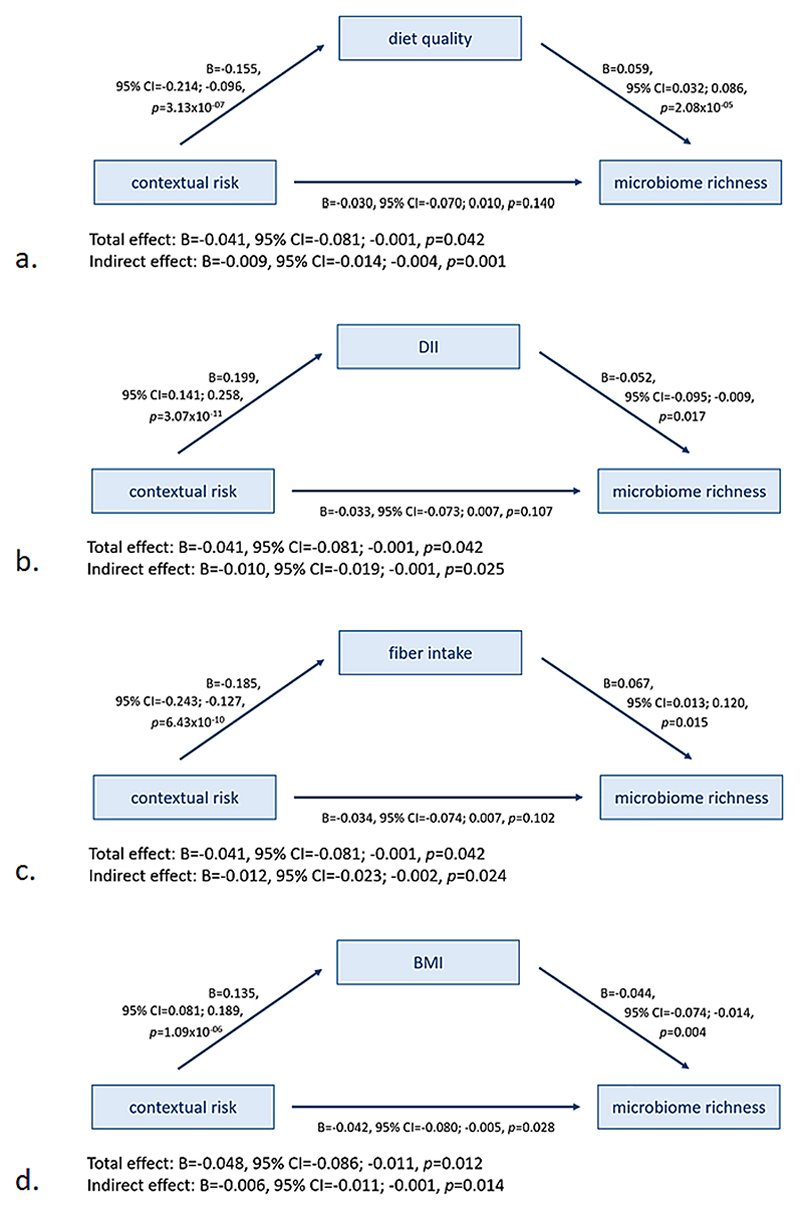
Mediation association of contextual risk and α-diversity measure microbiome richness by (a) diet quality, (b) Dietary Inflammatory Index (DII), (c) fiber intake, and (d) sex- and age-adjusted body mass index (BMI). The associations are adjusted for the other stress domains (life events, parental risk, interpersonal risk, direct victimization) and covariates (child sex, age, 5 genetic PCs, time in mail, season of production, batch, and number of reads). Results are pooled estimates from 30 imputed datasets.

The association between contextual risk and the α-diversity index microbiome richness was mediated by diet quality (indirect effect: B = −0.009, 95 %CI = −0.014; −0.004, *p* = 0.001), the Dietary Inflammatory Index (indirect effect: B = −0.010, 95 %CI = −0.019; −0.001, *p* = 0.025), and fiber intake (indirect effect: B = −0.012, 95 %CI = −0.023; −0.002, *p* = 0.024), and partially mediated by BMI SD (indirect effect: B = −0.006, 95 %CI = −0.011; −0.002, *p* = 0.014) (Fig. 4). The mediators diet quality, Dietary Inflammatory Index, and fiber intake rendered the direct effect no longer significant (*p* > 0.05). No evidence for mediation was found for total caloric intake, relative protein intake, or relative fat intake. For Shannon diversity, contextual risk was mediated by diet quality (indirect effect: B = −0.012, 95 %CI = −0.020; −0.005, *p* = 0.002; Supplementary Fig. 2), the Dietary Inflammatory Index (indirect effect: B = −0.017, 95 %CI = −0.030; −0.004, *p* = 0.013; Supplementary Fig. 3), and fiber intake (indirect effect: B = −0.016, 95 %CI = −0.031; −0.001, *p* = 0.038; Supplementary Fig. 4), each rendering the direct effect no longer significant, whereas the other variables did not emerge as significant mediators.

Last, associations of contextual risk with the L-tryptophan biosynthesis pathway was partially mediated by diet quality (indirect effect: B = −7.53 × 10^−06^, 95 %CI = −1.43 × 10^−05^; −7.93 × 10^−07^, *p* = 0.028; Supplementary Fig. 5) and the Dietary Inflammatory Index (indirect effect: B = −9.75 × 10^−06^, 95 %CI = −1.82 × 10^−05^; −1.31 × 10^−06^, *p* = 0.024; Supplementary Fig. 6). The inosine-5′-phosphate biosynthesis III pathway was partially mediated by diet quality (indirect effect: B = 8.30 10^−06^, 95 %CI = 1.51 × 10^−06^; 1.51 × 10^−05^, *p* = 0.017; Supplementary Fig. 7). The (R,R)-butanediol biosynthesis superpathway was partially mediated by the Dietary Inflammatory Index (indirect effect: B = −4.45 × 10^−06^, 95 %CI = −8.03 × 10^−06^; −8.58 × 10^−06^, *p* = 0.015; Supplementary Fig. 8) as well as by fiber intake (indirect effect: B = −3.98 × 10^−06^, 95 %CI = −7.33 × 10^−06^; −6.26 × 10^−07^, *p* = 0.020; Supplementary Fig. 9). There was no evidence for mediation by the other variables.

## 4 Discussion

In this study we investigated the association between ELS and the gut microbiome in a population-based sample of over 2,000 children. We examined associations with both overall ELS as well as five specific ELS domains, and studied the microbiome at four different levels of analysis. No associations were found between *overall* ELS and α-diversity, univariate genera abundances, β-diversity, or functional pathways after multiple testing correction. Of the five ELS domains investigated, only contextual risk showed independent associations with the gut microbiome. Associations of contextual risk, which includes stressors related to socio-economic disadvantage, were found at different levels of the microbiome, as it was associated with lower richness and lower Shannon diversity, with β-diversity, and differential enrichment of multiple athways, including the L-tryptophan biosynthesis pathway, the inosine-5′-phosphate biosynthesis III pathway, and the (R,R)-butanediol biosynthesis superpathway. These associations were largely mediated by dietary factors, such as diet quality, fiber intake and dietary pro-inflammatory potential, as well as BMI, which may point to potentially modifiable targets for enhancing the gut ecosystem and its functions in children exposed to contextual risk factors.

Our findings suggest that children who are exposed to higher contextual risk from birth to age 10, show a less diverse gut microbiome. Typically, a lower alpha diversity index is related to poorer gut health, as indicated by its associations with a poorer diet (Bowyer et al., 2018; Sweeney and Morton, 2013), obesity (Pinart et al., 2021; Yu et al., 2021), and inflammation (Clemente et al., 2018; Wang et al., 2022). Contextual risk was also associated with differences in the relative composition of the microbiota, as indicated by differences in β-diversity, but specific taxa contributing to the differences could not be identified as the differential abundance analysis did not show significant associations for any of the genera. This is in contrast to the results of several other studies on ELS and the microbiome (Callaghan et al., 2020; Flannery et al., 2020; Hantsoo et al., 2019; Michels et al., 2019; Reid et al., 2021), which have reported differential abundance of multiple genera − or taxa within genera − including *Bacteroides* (Flannery et al., 2020; Michels et al., 2019; Reid et al., 2021), *Prevotella* (Hantsoo et al., 2019; Reid et al., 2021), and *Streptococcus* (Flannery et al., 2020; Reid et al., 2021). However, given that these genera include a relatively high amount of bacterial species and ASVs and several ELS studies tested microbiome abundances at ASV- or species level (Flannery et al., 2020; Reid et al., 2021), it is perhaps unsurprising that these came up more often. In contrast, in our study, we focused on the genus-level, since counts would be unreliably low at lower levels. Other potential explanations for discrepant findings include differences in methodology (sample storage and processing, 16S rRNA region amplified, etc.), study population (e.g., selected based on a specific exposure versus population-based), sample size and statistical power, and different conceptualizations of ELS (e.g., focus on exposures such as adoption from orphanages vs the contextual risks examined here). Together, these differences render direct comparison between studies challenging.

Last, we found several predicted functional pathways to be associated with contextual risk, which could indicate taxonomic differences in the overall microbiota structure as reflected in the β-diversity differences. One of these pathways is involved in L-tryptophan biosynthesis. Tryptophan is the precursor to serotonin, a key neurotransmitter implicated in mood and typically targeted by antidepressants − which, unlike serotonin, can cross the blood−brain barrier to the central nervous system and plays a role in modulation of both peripheral and central neuroinflammatory responses (Mithaiwala et al., 2021; Seo and Kwon, 2023). This finding is consistent with a previous study that found socio-economic risk in children to be associated with microbiome functional pathways of monoamine metabolism, including a tryptophan metabolism pathway. This pathway was enriched for children exposed to more stressful life events, and specifically to life events related to family separation or social service involvement and to family illness or injury (Flannery et al., 2020). Together with our findings, this could suggest a potential mechanism through which contextual risk may be related to risk for mood disorders such as depression. However, we note that a recent study in the Generation R Study population did not show associations between the gut microbiome and depressive symptoms at age 10 years after multiple testing correction (Kraaij et al., 2023), potentially due to the early age examined. It will be important to follow up these results longitudinally to test whether differences in the L-tryptophan biosynthesis pathway during childhood may mediate ELS effects on depressive symptoms in late adolescence or early adulthood − a key period for the onset of depressive disorders (Richards, 2011). Further, in a large scale study of the human gut microbiome and inflammation, microbial tryptophan metabolism was found to influence cytokine production (Schirmer et al., 2016), suggesting that this is a mechanism through which the microbiome influences immune functioning. Another pathway enriched in relation to contextual risk was the inosine-5′-phosphate biosynthesis III pathway. This pathway is involved in the biosynthesis of purine nucleosides, building blocks of DNA and RNA. Gut-derived inosine has been shown to be a predictor of immunotherapy effectiveness (Mager et al., 2020). Lower inosine has also been related to major depressive disorder (Ali-Sisto et al., 2016; Zhou et al., 2019) although our findings point to an inverse relationship with ELS. Last, contextual risk was found to be associated with the super-pathway of (R,R)-butanediol biosynthesis. Butanediol is an alcohol and this pathway has been related to the intake of fermented dairy products, such as yoghurt (Bolte et al., 2021). It may be that (parents of) participants experiencing more contextual risk have a different diet, which is reflected in their gut microbiome.

Related to this, the contextual risk score was built to tap into socio-economic stress, including risk factors such as (persistent) financial difficulties, neighborhood problems and low maternal education. Since this was the only domain found to be associated with the microbiome, it may be that associations detected are more indicative of practical challenges related to socio-economic disadvantage, such as less healthy food options, poorer housing, or disease exposure, rather than the experience of stress itself. For example, in Rotterdam, the Netherlands, where the Generation R Study is based, it was found that socioeconomically disadvantaged areas have over 20 % more unhealthy food locations compared to other areas, with this discrepancy widening even more in recent years (Molenberg et al., 2019). Further, in industrialized countries, lower socio-economic status is consistently related to obesity (Wang and Lim, 2012). Consistent with this, our exploratory analyses showed that associations between contextual risk and the microbiome were at least in part mediated by dietary factors and BMI. On the other hand, we did not find a significant mediation effect through the inflammatory marker CRP. While it could be that inflammation does not play a role in this association, there may also be other explanations, including the reduced sample size for this mediation analysis, the fact that CRP has a short half-life of only 19 h, meaning it is mainly a measure of acute inflammation, and potential healthy bias arising from the fact that that children are less likely to visit the research center when sick. Indeed, we did find that more contextual risk was related to a higher dietary pro-inflammatory potential, as well as lower diet quality, lower fiber intake, and higher BMI, and that these in turn were related to a lower microbiome diversity. Dietary fibers are indigestible carbohydrates, which are the main source of energy for the gut bacteria. As such, dietary fibers promote the immunocompetence of the microbiome (Kuo, 2013; Nicolucci et al., 2015), and are an important component of the Dietary Inflammatory Index (Shivappa et al., 2014). According to the Dutch national guidelines (Health Council of the Netherlands, 2006), the current study population should be eating over 3.0−3.2 g/MJ of dietary fiber per day, depending on their age, which is higher than the average of 2.7 g/MJ/day reported here. Specifically, the diets of only 25 % of children adhered to this guideline, leaving ample room for improvement. Taken together, these results suggest that targeting modifiable factors such as fiber intake may dampen the negative effects of socioeconomic stress and disadvantage on the gut ecosystem, with potential downstream benefits for mental and physical health.

This study has several limitations. First, similar to other studies on ELS and the microbiome (Callaghan et al., 2020; Coley et al., 2021; Hantsoo et al., 2019; Michels et al., 2019; Reid et al., 2021), we utilized 16S rRNA sequencing instead of shotgun metagenomics sequencing. Metagenomic sequencing provides more detailed taxonomic and functional information than 16S rRNA sequencing (Laudadio et al., 2018), thus it is possible that the current method was not sensitive enough to detect some ELS-microbiome associations. To partly bridge this gap, we used an 16S rRNA pipeline including PICRUSt2 for functional pathway prediction, which has been shown to produce results that approach those of metagenomic sequencing (Langille et al., 2013). Second, we note that the identified effects are small, particularly the proportion of shared variance between contextual risk and β-diversity. To an extent, small effect sizes are inherent to population-based research, where interindividual differences are not as pronounced as, for example, in case-control designs. However, this approach gives us the opportunity to characterize associations within the general population using a long-term follow-up design, while additionally adjusting for relevant confounders, as well as to study potential mediators. Third, an inflammatory marker more adept to gauge chronic low-grade inflammation, such as GlycA, might have been more informative (Mokkala et al., 2020), but was not available in this study. Last, our exploratory follow-up mediation analyses focused on one mediator at a time and mediators correlated with each other. Given this set-up, we do not know if, for example, the pro-inflammatory potential of the diet mediated the associations over overall diet quality. Moreover, it is possible that several diet factors jointly mediate the contextual risk − microbiome association, that diet and BMI serially mediate this association. Furthermore, it is quite likely that associations between the microbiome and diet, BMI, and inflammation are bidirectional. However, these exploratory analyses can help to generate hypotheses for advanced testing to better understand how these factors relate over time.

Our analyses suggest that diet quality, fiber intake, and BMI may be relevant mediators of contextual risk and the microbiome. Future studies are needed, however, to establish the directionality and causality of the identified associations between contextual risk, the microbiome, and aforementioned mediators, for example through advanced epidemiological methods to infer causality, such as Mendelian randomization, provided that robust genetic instruments are available. The use of dietary interventions in experimental studies could also be considered. An earlier mouse study, for example, showed that prebiotic administration could reverse the effect of chronic stress on the microbiome, increase L-tryptophan levels, and reduce depressive and anxious behaviors (Burokas et al., 2017), although it is currently unclear whether similar findings extend to humans. Finally, based on previous literature (Bowyer et al., 2018; Clemente et al., 2018; Pinart et al., 2021; Sweeney and Morton, 2013; Wang et al., 2022; Yu et al., 2021), we interpret our findings of a lower microbiome diversity as indicative of poorer gut health, but more research is needed to understand downstream effects on ELS-associated microbiome changes and enriched functional pathways on later physical and mental health.

In conclusion, this is the first population-based study investigating the links between early-life stress and the microbiome. We find limited evidence for associations between overall or specific domains of earlylife stress and the microbiome. We do, however, find that contextual risk, which is reflective of socio-economic stress, associates with differences in children’s microbiome at the level of diversity, composition and related functional pathways. Exploratory analyses indicate that these associations may be mediated by factors related to food intake and BMI. Future studies are needed to infer causality and establish whether these modifiable factors could be targeted to enhance gut health, especially in children exposed to more contextual stress.

## Supplementary Material

Supplementary figures

Supplementary tables

## Figures and Tables

**Table 1:** Sample descriptives.

*N*	2004
Age, y (mean (SD))	9.8 (0.3)
Sex, boys *(n* (%))	1010 (50.4)
Overall early-life stress score	0.75 (0.47)
Life events score	0.24 (0.14)
Contextual risk score	0.22 (0.21)
Parental risk score	0.08 (0.13)
Interpersonal risk score	0.09 (0.13
Direct victimization score	0.11 (0.13)
Time stool sample took to reach the lab (time in mail), d (mean (SD))	2.4 (1.2)
Antibiotic use (*n* (%))	
No antibiotic use in the last year	1846 (92.1)
Within the past month	30 (1.5)
1 to 3 months ago	43 (2.1)
3 months to a year ago	81 (4.0)
missing	4 (0.2)
Parental national origin (*n* (%))	
Dutch	1245 (62.1)
European (non-Dutch, non-Turkish)	153 (7.6)
Turkish	99 (4.9)
Moroccan	78 (3.9)
Surinamese	117 (5.7)
Indonesian	16 (0.8)
Dutch Caribbean	48 (2.4)
Other African	105 (5.2)
Other American	57 (2.8)
Other Asian	57 (2.8)
Oceanian	5 (0.2)
missing	24 (1.2)
Maternal education (*n* (%))	
Lower (no education finished, primary school finished)	111 (5.5)
Mid (secondary school finished)	742 (37.0)
Higher (higher education finished)	1008 (50.3)
missing	143 (7.1)
*n*	1,594
Diet quality score at age 8 years (mean (SD))	4.54 (1.23)
Dietary Inflammatory Index (mean (SD))	−0.34 (0.78)
Total energy intake, kcal (mean (SD))	1496 (380)
Protein intake, E% (mean (SD))	16.5 (2.25)
Fat intake, E% (mean (SD))	32.5 (4.42)
Carbohydrate intake, E% (mean (SD))	51.0 (5.0)
Dietary fiber intake, gram/MJ (mean (SD))	2.7 (0.6)
*n*	2,003
BMI at age 10, kg/m^2^ (mean (SD))	17.3 (2.5)
*n*	1,420
C-reactive protein (*n* (%))	
Normal, 0−1 mg/L	1,163
Low grade inflammation, 1−3 mg/L	177
Moderate to severe inflammation, >3 mg/L	80

**Table 2:** Associations between early-life stress and α-diversity.

	β (95 % CI)	*p*-value	*q*-value
*Overall early-life stress*			
Richness	0.003 (−0.029; 0.035)	0.872	0.872
Shannon diversity	− 0.017 (−0.062; 0.029)	0.472	0.707
inverse Simpson diversity	− 0.031 (−0.077; 0.016)	0.194	0.581
*Life events*			
Richness	0.001 (−0.032; 0.029)	0.939	0.963
Shannon diversity	0.009 (−0.035; 0.053)	0.685	0.963
inverse Simpson diversity	− 0.001 (−0.046; 0.044)	0.963	0.963
*Contextual risk*			
Richness	− 0.048 (−0.086; − 0.011)	0.010	**0.031**
Shannon diversity	− 0.063 (−0.116; − 0.010)	0.021	**0.031**
inverse Simpson diversity	− 0.051 (−0.106; 0.003)	0.064	0.064
*Parental risk*			
Richness	0.032 (0.000; 0.063)	0.048	0.079
Shannon diversity	0.045 (−0.000; 0.090)	0.053	0.079
inverse Simpson diversity	0.036 (−0.010; 0.082)	0.122	0.122
*Interpersonal risk*			
Richness	0.023 (−0.011; 0.057)	0.190	0.571
Shannon diversity	− 0.004 (−0.053; 0.045)	0.881	0.881
inverse Simpson diversity	− 0.015 (−0.065; 0.035)	0.549	0.823
*Direct victimization*			
Richness	− 0.002 (−0.034; 0.029)	0.889	0.889
Shannon diversity	− 0.011 (−0.056; 0.034)	0.623	0.889
inverse Simpson diversity	− 0.015 (−0.061; 0.031)	0.520	0.889

Models were adjusted for child sex, age, 5 genetic PCs, the time in mail, season of production, batch (DNA Isolation batch and sequencing batch), and number of reads. In addition, models with the risk domains (life events, contextual risk, parental risk, interpersonal risk, and direct victimization) were mutually adjusted for the other risk domains. Q-values are FDR-adjusted within each stressor, over the three α-diversity indices. Results are pooled estimates from 30 imputed datasets.

**Table 3:** Associations between specific stress domains and β-diversity.

	median F (IQR)	median R^2^ (IQR)	median *p*-value (IQR)
Overall early-life stress	1.40 (1.37; 1.43)	0.0007 (0.0007; 0.0007)	0.057 (0.044; 0.067)
Life events	1.41 (1.40; 1.42)	0.0007 (0.0007; 0.0007)	0.055 (0.051; 0.060)
Contextual risk	2.33 (2.29; 2.37)	0.0011 (0.0011; 0.0012)	**0.001 (0.001;** **0.001)**
Parental risk	1.38 (1.37; 1.39)	0.0007 (0.0007; 0.0007)	0.066 (0.058;0.071)
Interpersonal risk	0.95 (0.93; 0.96)	0.0005 (0.0004; 0.0004)	0.545 (0.504; 0.574)
Direct victimization	0.83 (0.83; 0.84)	0.0004 (0.0004; 0.0004)	0.755 (0.744; 0.775)

Models were adjusted for child sex, age, 5 genetic PCs, time in mail, season of production, and batch (DNA Isolation batch and sequencing batch). Median (IQR) F, R^2^ and *p*-values represent the median and interquartile range result values over 30 imputed sets.

**Table 4:** Associations between contextual risk and functional microbial pathways.

MetaCyc pathway	Description	B (95 % CI)	*p-*value	*q-* value
TRPSYN- PWY	L-tryptophan biosynthesis	−1.05 × 10^−04^ (−1.49 × 10^−04^; −6.07 × 10^−05^)	3.66 × 10^−06^	0.002
PWY−7234	inosine-5′-phosphate biosynthesis III	8.88 × 10^−05^ (4.48 × 10^−05^; 1.33 × 10^−04^)	8.06 × 10^−05^	0.032
P125-PWY	superpathway of (R,R)- butanediol biosynthesis	−3.27 × 10^−05^ (−5.06 × 10^−05^; −1.48 × 10^−05^)	3.61 × 10^−04^	0.047

Models were adjusted for child sex, age, 5 genetic PCs, the time in mail, season of production, batch (DNA Isolation batch and sequencing batch), and number of reads. In addition, models with the risk domains (life events, contextual risk, parental risk, interpersonal risk, and direct victimization) were mutually adjusted for the other risk domains. Q-values are FDR-adjusted within each stressor, over the 309 constructed pathways. Results are pooled estimates from 30 imputed datasets.

## Data Availability

Data from this study are available upon reasonable request to the director of the Generation R Study (generationr@erasmusmc.nl), subject to local, national and European rules and regulations.
