# Supplementary material for: Early-life stress and the gut microbiome: A comprehensive population-based investigation
Source: Brain Behav Immun. Author manuscript; Available in PMC 2024 Apr 3. (PMC7615798; doi:10.1016/j.bbi.2024.02.024)
Supplement: Supplementary figures [file EMS194683-supplement-Supplementary_figures.docx]

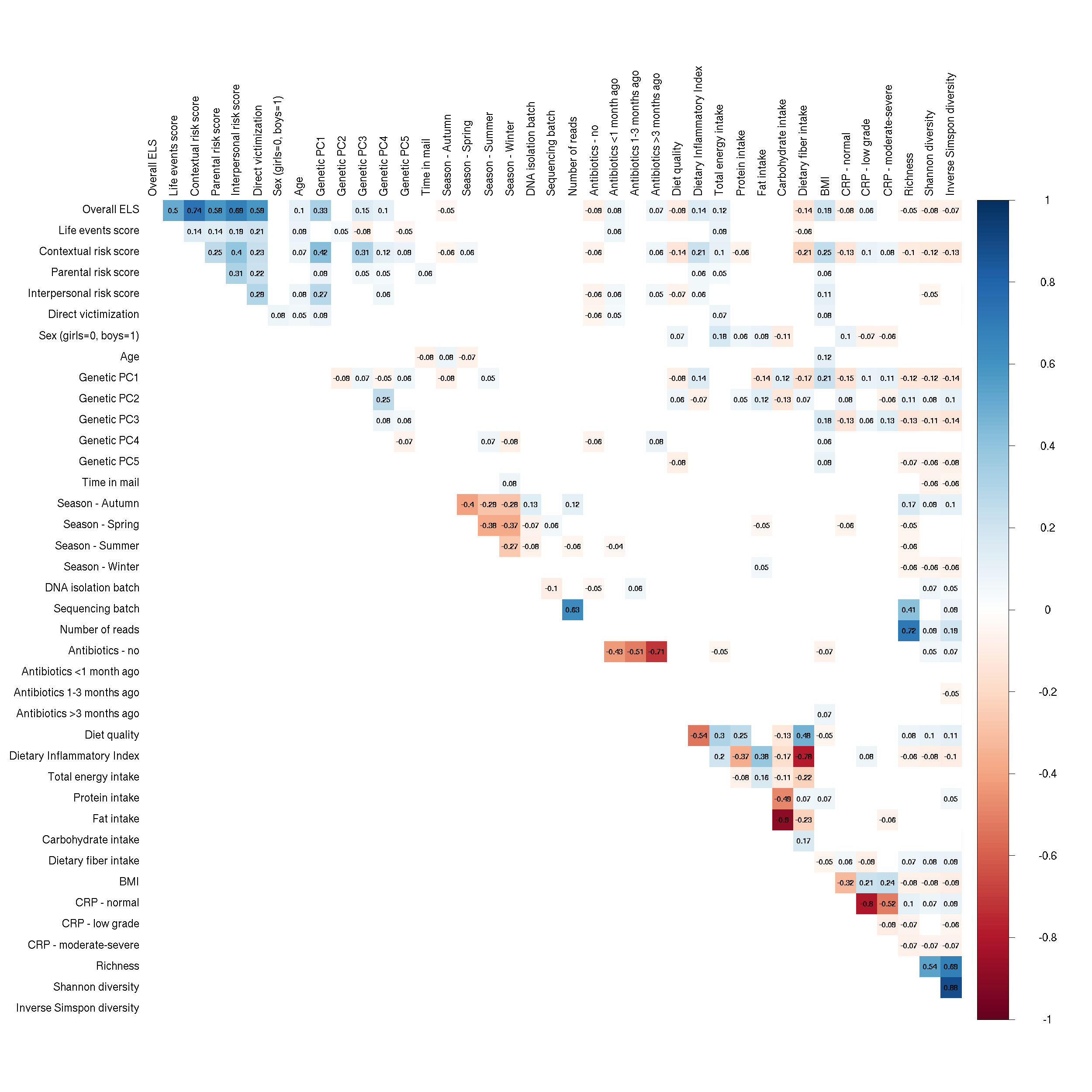


**Supplementary Figure 1.** Correlational plot of Pearson correlations of early-life stress measures, covariates, mediators, and alpha diversity measures. Only correlations of *p*<0.05 are depicted.


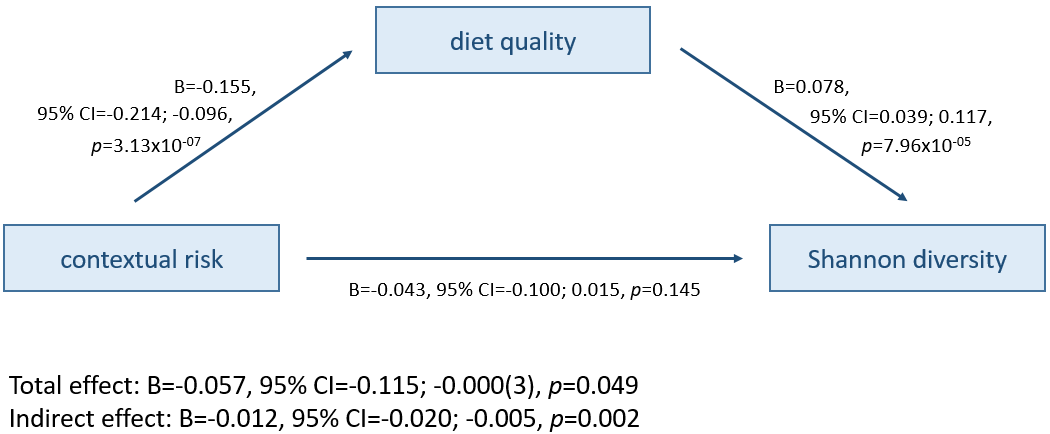


**Supplementary Figure 2.** Mediation association of contextual stress and Shannon diversity by diet quality. The associations are adjusted for the other stress domains (life events, parental risk, interpersonal risk, direct victimization) and covariates (child sex, age, 5 genetic PCs, time in mail, season of production, batch, and number of reads). Results are pooled estimates from 30 imputed datasets.


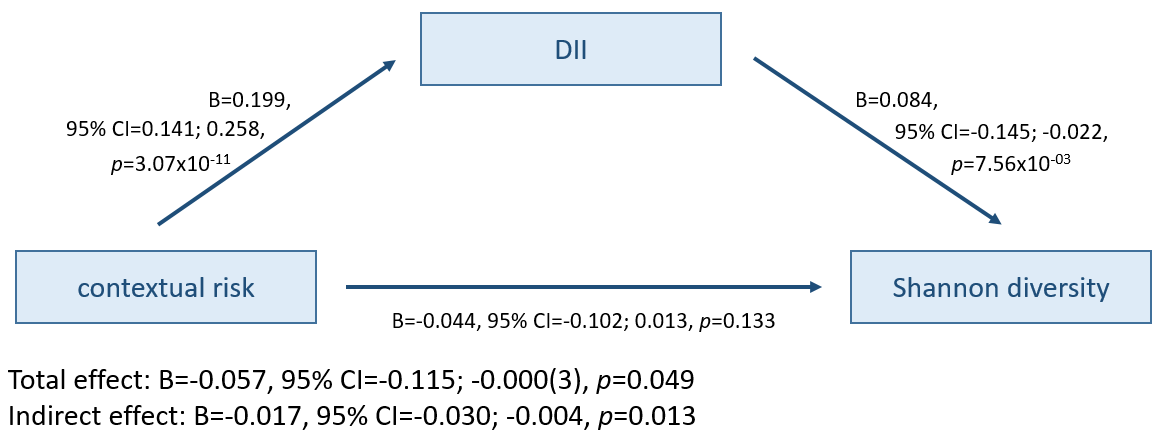


**Supplementary Figure 3.** Mediation association of contextual stress and Shannon diversity by the Dietary Inflammatory Index (DII). The associations are adjusted for the other stress domains (life events, parental risk, interpersonal risk, direct victimization) and covariates (child sex, age, 5 genetic PCs, time in mail, season of production, batch, and number of reads). Results are pooled estimates from 30 imputed datasets.

**
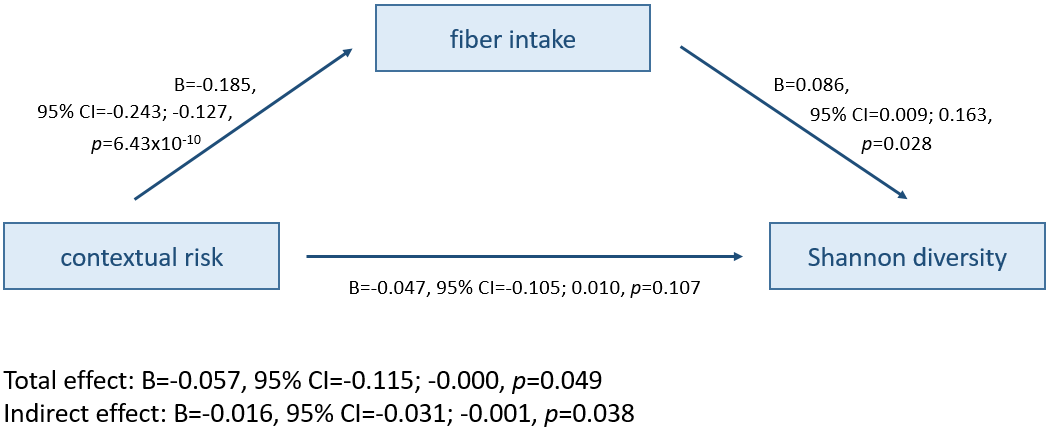
**

**Supplementary Figure 4.** Mediation association of contextual stress and Shannon diversity by fiber intake. The associations are adjusted for the other stress domains (life events, parental risk, interpersonal risk, direct victimization) and covariates (child sex, age, 5 genetic PCs, time in mail, season of production, batch, and number of reads). Results are pooled estimates from 30 imputed datasets.


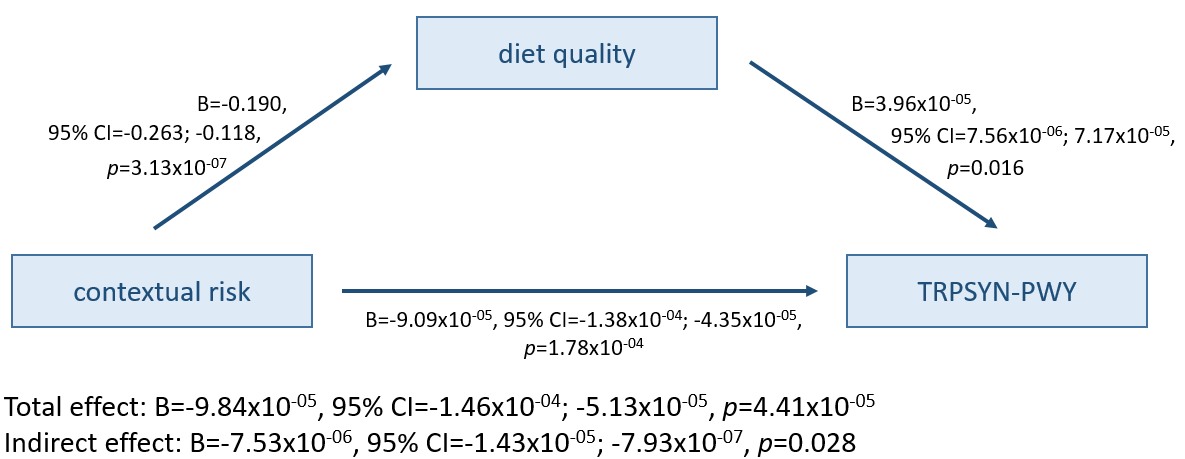


**Supplementary Figure 5.** Mediation association of contextual stress and L-tryptophan biosynthesis pathway TRPSYN-PWY by diet quality. The associations are adjusted for the other stress domains (life events, parental risk, interpersonal risk, direct victimization) and covariates (child sex, age, 5 genetic PCs, time in mail, season of production, batch, and number of reads). Results are pooled estimates from 30 imputed datasets.


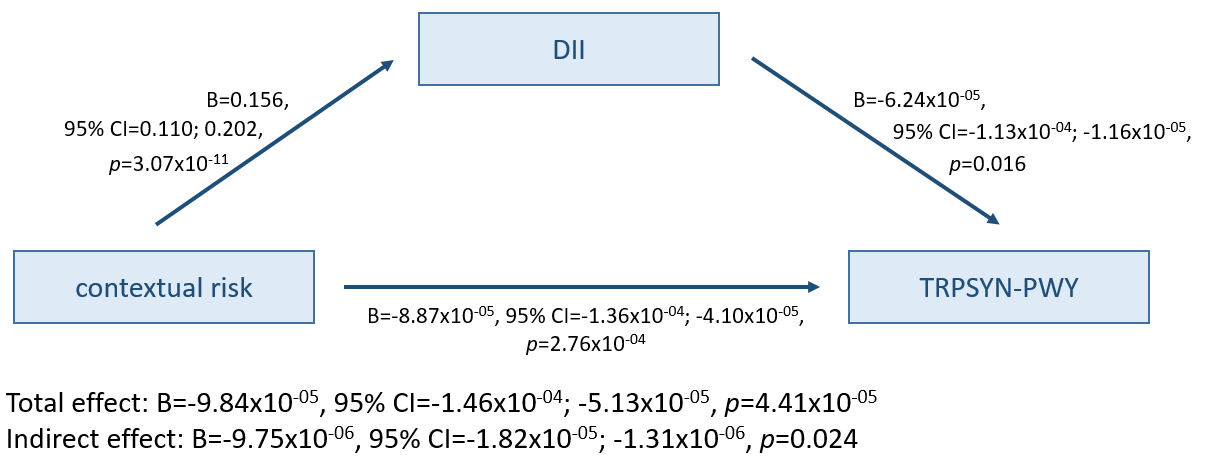


**Supplementary Figure 6.** Mediation association of contextual stress and L-tryptophan biosynthesis pathway TRPSYN-PWY by the Dietary Inflammatory Index (DII). The associations are adjusted for the other stress domains (life events, parental risk, interpersonal risk, direct victimization) and covariates (child sex, age, 5 genetic PCs, time in mail, season of production, batch, and number of reads). Results are pooled estimates from 30 imputed datasets.


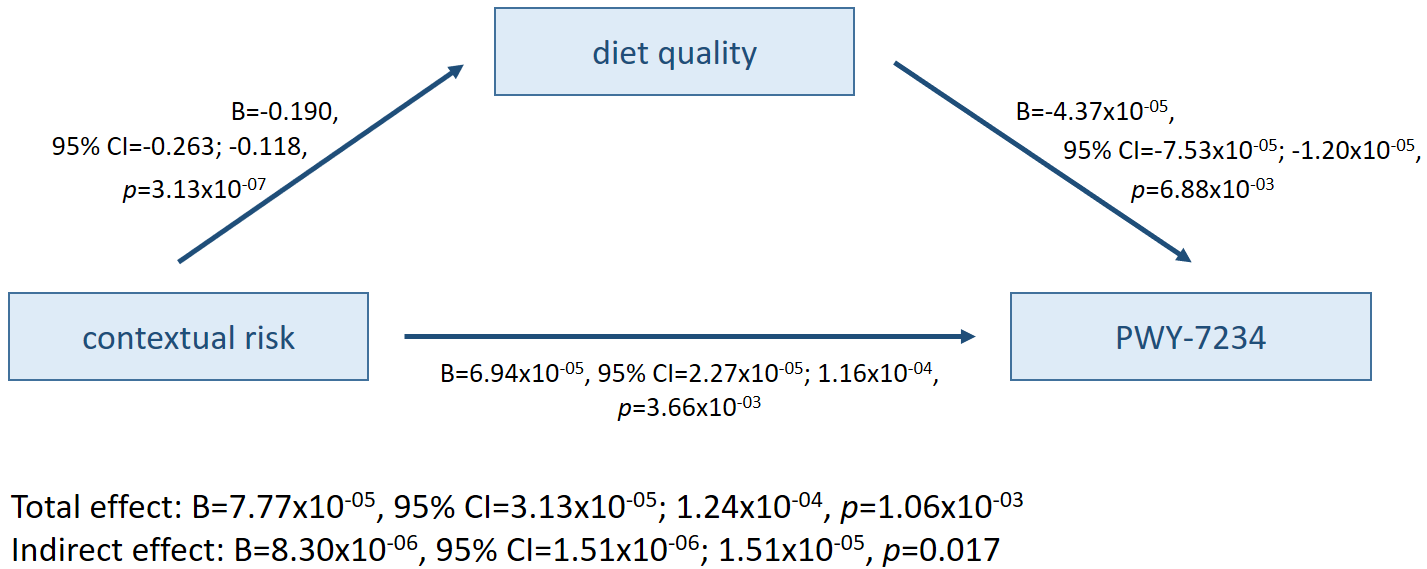


**Supplementary Figure 7.** Mediation association of contextual stress and inosine-5’-phosphate biosynthesis III pathway PWY-7234 by diet quality. The associations are adjusted for the other stress domains (life events, parental risk, interpersonal risk, direct victimization) and covariates (child sex, age, 5 genetic PCs, time in mail, season of production, batch, and number of reads). Results are pooled estimates from 30 imputed datasets.

**
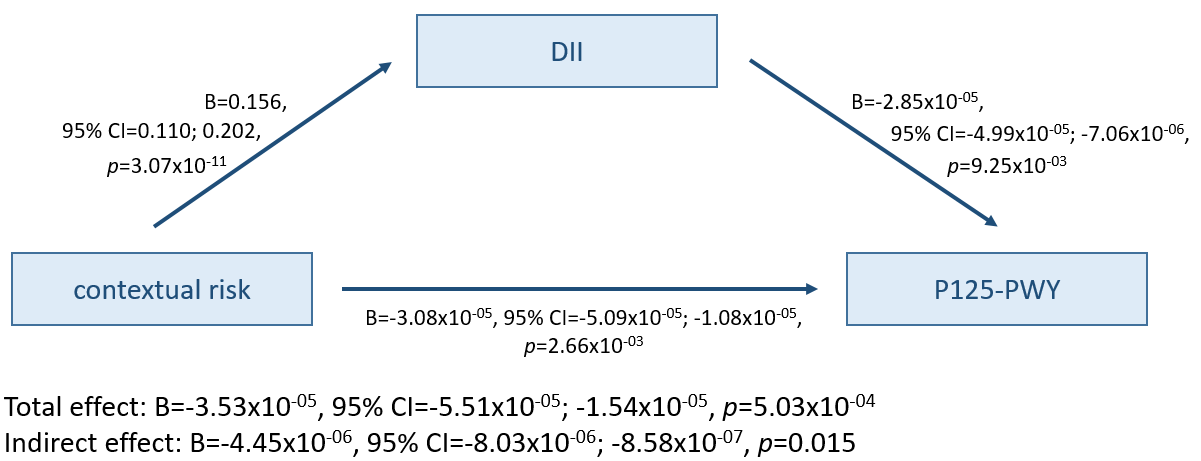
**

**Supplementary Figure 8.** Mediation association of contextual stress and (R,R)-butanediol biosynthesis superpathway P125.PWY by the Dietary Inflammatory Index (DII). The associations are adjusted for the other stress domains (life events, parental risk, interpersonal risk, direct victimization) and covariates (child sex, age, 5 genetic PCs, time in mail, season of production, batch, and number of reads). Results are pooled estimates from 30 imputed datasets.


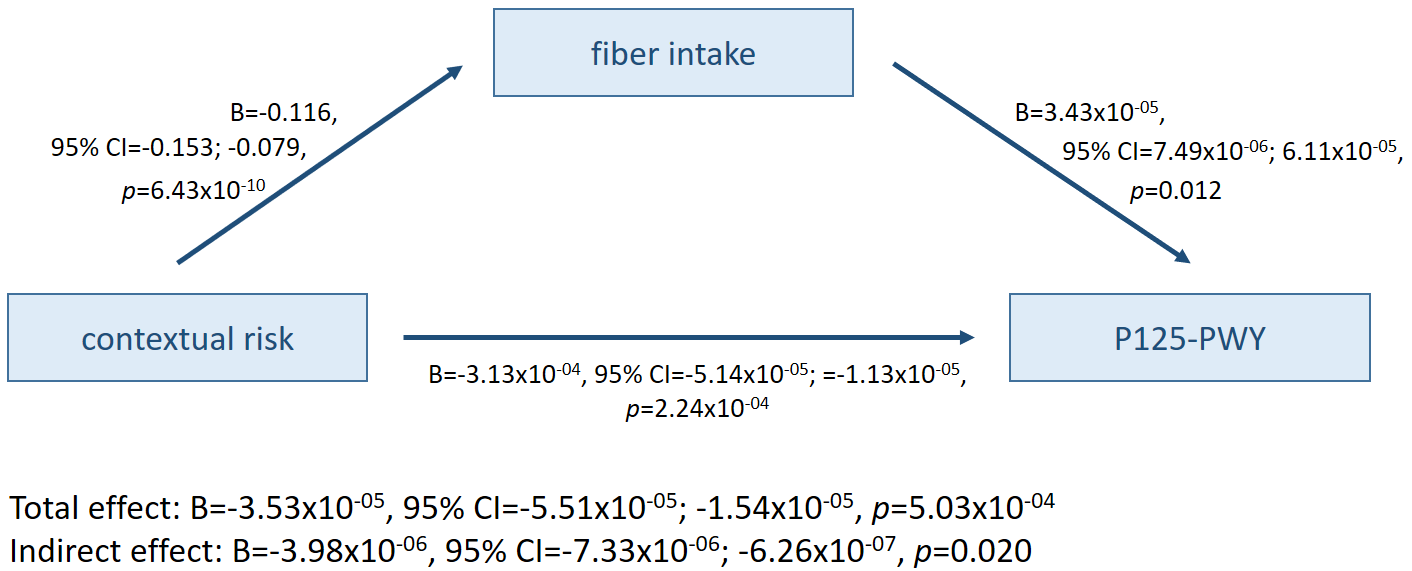


**Supplementary Figure 9.** Mediation association of contextual stress and (R,R)-butanediol biosynthesis superpathway P125.PWY by fiber intake. The associations are adjusted for the other stress domains (life events, parental risk, interpersonal risk, direct victimization) and covariates (child sex, age, 5 genetic PCs, time in mail, season of production, batch, and number of reads). Results are pooled estimates from 30 imputed datasets.
